# Associations of major dietary patterns with cardiometabolic risk factors among Iranian patients with type 1 diabetes

**DOI:** 10.1016/j.pmedr.2024.102618

**Published:** 2024-01-18

**Authors:** Zahra Shojaeian, Zohreh Ebrahimi, Fatemehsadat Amiri, Ahmad Esmaillzadeh, Omid Sadeghi, Seyed Adel Jahed, Alireza Esteghamati, Ali Ebrahimkhani

**Affiliations:** a Department of Nutrition, School of Public Health, Iran University of Medical Sciences, Tehran, Iran; b Department of Community Nutrition, School of Nutritional Sciences & Dietetics, Tehran University of Medical Sciences, Iran; c Nutrition and Food Security Research Center, Department of Community Nutrition, Student Research Committee, School of Nutrition and Food Science, Isfahan University of Medical Sciences, Isfahan, Iran; d Gabric Diabetes Education Association, Tehran, Iran; e Endocrinology and Metabolism Research Center (EMRC), Internal Medicine Department, Vali-Asr Hospital, Tehran University of Medical Sciences, Tehran, Iran

**Keywords:** Type 1 diabetes, Dietary patterns, Cardiometabolic risk factors, Factor analysis

## Abstract

**Objective:**

To examine the associations between dietary patterns and cardiometabolic risk factors among type 1 diabetic (T1D) patients.

**Methods:**

This cross-sectional study was conducted on 229 Iranian T1D patients. Data on dietary intakes were collected using a 168-item food frequency questionnaire. To identify major dietary patterns, we merged data on the 168 food items to obtain 23 food groups. Then, we constructed major dietary patterns using factor analysis with varimax rotation. We used binary logistic regression to assess the association between dietary patterns and cardiometabolic risk factors, in which potential confounders were adjusted.

**Results:**

Four dietary patterns were identified: Western, unhealthy, traditional, and semi-healthy patterns. After adjusting for confounders including demographic variables, physical activity, energy intake, and medical history, participants in the highest tertile of the Western dietary pattern had 2.53 (95 % CI: 1.03–6.22) and 3.37 (95 % CI: 1.18–9.63) times more odds of elevated HbA1c and low estimated glucose disposal rate (eGDR), respectively, compared with those in the lowest tertile. Such the positive association was also seen for elevated fasting blood glucose (FBG). Moreover, individuals in the top tertile of unhealthy diet had more odds of elevated LDL-c and abdominal obesity than those in the lowest tertile. Regarding the semi-healthy diet, higher adherence was associated with 51 % lower odds of elevated FBG (OR: 0.49, 95 % CI: 0.24–0.99). For other outcomes, no significant association was found.

**Conclusion:**

We found that T1D patients may take benefit from adherence to a semi-healthy diet with a low amount of unhealthy and Western-related foods.

## 1 Introduction

Cardiometabolic risk factors including dyslipidemia, general and abdominal obesity, elevated blood pressure, insulin resistance, and abnormal glucose homeostasis are the main complications of diabetes mellitus which increase the risk of mortality among these patients (Danaei et al., 2014, Mudaliar et al., 2016). The management and prevention of these risk factors can promote quality of life, control diabetes symptoms, and prevent early mortality in diabetic patients (Secrest et al., 2010, Cleland, 2012).

Diet plays an important role in the incidence of cardiovascular disease (CVD) or their risk factors (AlAufi et al., 2022, Agodi et al., 2018, Snell-Bergeon et al., 2009, Olinto et al., 2012). However, current data on diet-CVD relationships are mostly from studies on type 2 diabetic (T2D) patients or general populations, and data on T1D are scarce (Barnard et al., 2006, Vitale et al., 2018). Previous studies have shown that higher intakes of fast-foods, calorie-dense foods, high-fat dairy products, and low intakes of vegetables, fruits, legumes, and nuts are associated with an increased risk of abdominal obesity, insulin resistance, hypertension, dyslipidemia, and abnormal glucose homeostasis which are known as cardiometabolic risk factors (Kahleova et al., 2019, Miri et al., 2018). However, it is not clear whether there are these associations among T1D patients or not. Since abnormal glucose homeostasis and other cardiometabolic risk factors are more prevalent among T1D patients compared with other populations, diet can play an important role in the progression and prevention of these risk factors (Leroux et al., 2014).

It should be kept in mind that people consume a combination of foods in the context of a dietary pattern. Therefore, the dietary pattern approach in assessing diet-disease relations is better than focusing on a single food or nutrient intake due to decreasing the co-linearity problem which might occur when assessing single food and nutrient intakes (Hu, 2002). Furthermore, in the dietary pattern approach, we can assess the synergic effects of foods on cardiometabolic risk factors. Also, the dietary patterns approach is the best method for assessing the associations of overall dietary quality with cardiometabolic risk factors (Ocké, 2013).

Prior studies revealed that adherence to healthy dietary patterns including the Mediterranean diet and DASH diet was associated with a reduced risk of CVDs or their risk factors (Kahleova et al., 2019). We are aware of no study that examined the relationship between major dietary patterns and cardiometabolic risk factors among T1D patients in the Middle East where people have a different dietary pattern compared with the Western population. Also, the prevalence of CVD among people with diabetes is estimated to be high in the Middle East (Bonakdaran et al., 2011). Therefore, the current study was conducted to examine the associations between major dietary patterns and cardiometabolic risk factors among Iranian T1D patients.

## 2 Materials and methods

### 2.1 Participants

This cross-sectional study was conducted on T1D patients whose information was registered at the Gabric Diabetes Education Association (GDEA) database and also databases from seven diabetes clinics located in Tehran, Iran, and the patient registry of Imam Khomeini hospital, Tehran, Iran. Patients registered at the mentioned databases were invited by letter to participate in the current study. We included patients with an age range of ≥18, those that had T1D for ≥1 year, patients who received insulin, and those who were willing to participate in the current study. Exclusion criteria included ≥1 severe hypoglycemic episode (requiring assistance) during the previous 3 months, pregnancy and lactation, advanced microvascular and macrovascular complications, hyperglycemia along with ketoacidosis in the past month, celiac disease, and having a weight loss program in the last year. Moreover, we excluded those patients who reported a total daily energy intake outside the range of 800–4200 kcal/d. After these exclusions, of 252 individuals who accepted our invitation, 229 remained for the current analysis. The study protocol was approved by the ethics committee of the Iran University of Medical Sciences, Tehran, Iran (IR.IUMS.REC 1395.9413468003). All participants provided informed written consent before participating in the current study.

### 2.2 Dietary intakes

Subjects were asked to report their usual dietary intakes via a validated 168-item semi-quantitative Food Frequency Questionnaire (FFQ) (Esfahani et al., 2010). Participants were asked to answer to this FFQ based on their dietary intakes during the past 12 months. To increase the precision and accuracy of estimates, two trained interviewers collected the dietary data. Daily nutrient intake was also calculated for each participant using the US Department of Agriculture (USDA) national nutrition database (Ahuja et al., 2012). The validity and reliability of the questionnaire were previously confirmed in a pilot study (Esfahani et al., 2010). In that study, the mean energy-adjusted correlation coefficients for overall nutrient intake between the 24-hour dietary recall and FFQ were between 0.37 and 0.44, indicating an appropriate reliability of the FFQ.

### 2.3 Biochemical assessment

Each participant provided a 12-mL venous blood sample after overnight fasting. Blood sampling was done using a vacuum needle (Greiner. Bio-One International Plus Needle 22G). Then, 2 ml of whole blood was stored in an ethylenediamine tetraacetic acid (EDTA) tube (Kima- Italy) at 4° C in the refrigerator, and after 2 h, hemoglobin A1c (HbA1c) was measured by a dedicated ion-exchange high-performance liquid chromatography instrument (TOSOH, Bioscience, Inc., San Francisco, CA). The remaining blood sample was allowed to clot (15 min), and then, was centrifuged at 5000 rpm for serum extraction. The serum sample was fractioned into different aliquots that were stored in freezers (–80 °C) for future assessments. Fasting blood glucose (FBG) and blood lipids including total cholesterol (TC), low-density lipoprotein cholesterol (LDL-c), high-density lipoprotein cholesterol (HDL-c), and triglycerides (TG) were measured using an enzymatic assay (Dialab Kits, Australia) that was performed by an auto-analyzer (BT1500; Biotecnica Instrument, Italy). All biochemical assessments were conducted in the laboratory of Pars hospital, Tehran, Iran.

### 2.4 Anthropometric measurements

Weight was measured to the nearest 0.1 kg using a Seca 807 digital scale while the participants had minimal clothing and no shoes. Standing height was measured without shoes using a wall-mounted stadiometer (Seca 206 standard stadiometer) to the nearest 0.1 cm when patients’ shoulders were at the stadiometer. Body mass index was calculated as weight (kg) divided by height squared (m^2^). Waist and hip circumferences (WC and HC) were measured to the nearest 0.5 cm (WC using a flexible tape at the midpoint between the last rib and the iliac crest at minimal respiration and HC on the widest point of the hip when the participant was in a standing position). The Waist-to-hip ratio (WHR) was also calculated. Body fat percentage (BFP) was measured using a body composition analyzer (Omron BF511, Germany). To reduce measurement bias in anthropometric data, measurements were done in the morning, after sample collection, while the participants were still fasting.

### 2.5 Insulin resistance assessment

Insulin resistance was determined using the estimated glucose disposal rate (eGDR) formula which was proposed and validated by Williams and colleagues (Williams et al., 2000): 24.31 – [(12.22 – WHR) – (3.29 – hypertension) – (0.57 – HbA1c)]. In this formula, the score of hypertension was 1 (blood pressure ≥140/90 mmHg or the use of antihypertensive medication) or 0 (<140/90 mmHg without antihypertensive medication). A higher eGDR value was associated with higher insulin sensitivity.

### 2.6 Assessment of other variables

Information on age, gender (male/female), education (university /lower than university), marital status (married/single), smoking (never or ex-smoker/current smoker), duration of diabetes (>10 y/<10 y), diabetes-related training (yes/no), and drug use (yes/no) was collected with a face-to-face interview using a research-made questionnaire. To increase the accuracy of data collected for drug use, participants were asked to bring the drugs that they consumed currently. In the current study, an ex-smoker was defined as an individual who has smoked >100 cigarettes throughout their life but has not smoked in the last month (Organization, 2003). Current smokers were considered as those individuals who smoke cigarettes either daily or occasionally (Organization, 2003). To measure blood pressure, first, subjects were asked to rest for 15 min, and then, blood pressure was measured two times with a five-minute interval on the left arm using a digital blood pressure monitor (Omron, Model M3 Intellisense, Mannheim, Germany). In this study, systolic and diastolic blood pressure (SBP and DBP) were recorded as the average of the two measurements. We used the International Physical Activity Questionnaire (IPAQ) to assess participants᾿ physical activity through a face-to-face interview. IPAQ results were expressed as metabolic equivalents per week (MET-hour/week). The validity and reliability of IPAQ among Iranian population were assessed previously (Moghaddam et al., 2012).

### 2.7 Statistical analysis

To identify major dietary patterns, firstly, data on the 168 food items were merged to obtain 23 food groups. This merging was based on the similarity of the nutrient content of food items (Table 1**)**. We constructed major dietary patterns using factor analysis in which the factors were rotated by varimax rotation. Major patterns obtained in the analysis were defined based on the visual inspection of scree plot and the eigenvalues of ≥1.35. The eigenvalue represents the variance explained by a specific dietary pattern. It is a measure of how much of the variation in the data can be attributed to that particular pattern. Researchers often consider eigenvalues greater than 1 to determine the number of meaningful dietary patterns to retain (Montano and Jombart, 2017). In the current study, we considered the cut point of 1.35 for eigenvalue because the first four dietary patterns had an eigenvalue of ≥1.35. The fifth pattern had an eigenvalue of <1. The factor score for each pattern was calculated by summing the intakes of food groups or items weighted by their factor loadings. Accordingly, each participant received a factor score for each dietary pattern. This score showed the adherence of participants to each pattern.

**Table 1:** Demographic characteristics of Iranian T1D patients aged ≥18 years, 2017–2019.

Variables	Female (142)	Male (87)	All
Age (y)	31.00 ± 10.02	34.43 ± 12.12	32.31 ± 10.96
BMI (kg/m^2^)	24.93 ± 3.65	24.58 ± 3.87	24.80 ± 3.73
HbA1c (%)	8.23 ± 1.52	7.89 ± 1.58	8.10 ± 1.55
PA (MET-h/wk)	418.5 ± 389.5	588.7 ± 513.9	483.2 ± 447.4
Daily insulin dose (unit)	47.94 ± 13.89	60.05 ± 20.73	52.54 ± 17.78
DM duration (>10 y %)	41.9 (96)	27.1 (62)	69.0(158)
Insulin type %			
Lantus and NovoRapid	40.6 (93)	24.9 (57)	65.5 (150)
NPH and Regular	12.7 (29)	10.0 (23)	22.7 (52)
Lantus and Apidra	3.9 (9)	2.2 (5)	6.1 (14)
Levemir and NovoRapid	4.8 (11)	0.9 (2)	5.7 (13)
Drug use (yes %) ^a^	13.1 (30)	10.5 (24)	23.6 (54)
DM-related training (yes %)	54.6 (1 2 5)	29.3 (67)	83.8 (1 9 2)
Current smoker %	5.2 (12)	14.8 (34)	20.1 (46)
University educated %	40.6 (93)	23.6 (54)	64.2 (1 4 7)
Married %	27.5 (63)	15.7 (36)	43.2 (99)

Data are presented as mean ± SD or percent (N).

Abbreviation: BMI: body mass index, T1D: type 1 diabetes, DM: diabetes mellitus, PA: physical activity, NPH: neutral protamine hagedorn, HbA1c: hemoglobin A1c, MET: metabolic equivalent.

*Obtained from the independent-sample *t*-test or chi-squared test, where appropriate.

^a^ Considered as the use of lipid-lowering medication, antihypertensive medication, and Levothyroxine.

Participants were categorized based on the tertiles of dietary pattern scores. We used one-way analysis of variance (ANOVA) to determine the differences in continuous variables across the tertiles of dietary pattern scores. Also, the Chi-square test was used to assess the distribution of participants in terms of categorical variables across the tertiles of dietary pattern scores. We used multiple logistic regression in adjusted models to obtain odds ratios (ORs) and 95 % confidence intervals (CIs) for the associations between dietary patterns and cardiometabolic risk factors [i.e. FBG > 130 mg/dL, HbA1c > 7 %, TG > 150 mg/dL, TC > 200 mg/dL, LDL-c > 100 mg/dL, low HDL-c (<40 mg/dL in males and <50 mg/dL in females), overweight or obesity (BMI ≥ 25 kg/m^2^) (Care, 2019), elevated blood pressure (SBP ≥ 130 mmHg and DBP ≥ 85 mmHg) (Grundy et al., 2004), low eGDR (the first tertile of eGDR), abdominal obesity (WC > 90 cm or WHR > 0.95 in males and >0.90 in females) (Azizi et al., 2010), high BFP (>25 % in males and >39 % in females)] (Jabłonowska-Lietz et al., 2017). To find appropriate confounders, we first included all potential confounders (age, gender, energy intake, smoking, duration of diabetes, DM-related training, physical activity, drug use, and BMI) in an adjusted model, and then, those confounders that had a significant effect in that model (P-value < 0.10) were included in the final adjusted model. Therefore, different confounding variables were adjusted for each outcome. These confounders are indicated in Supplementary Table 1. In the analysis, individuals in the first tertile of dietary pattern scores were considered as a reference group. All analyses were performed using the SPSS software (version 18). P-values were considered significant at <0.05.

## 3 Results

In total, 229 T1D patients were included in the current analysis. Since the data collection was conducted by a face-to-face interview, we had complete data on exposure, outcome, and confounding variables. Mean age of study participants was 32.31 ± 10.96 years. Participants' demographic characteristics are shown in Table 2. Mean age of study participants was 32.31 ± 10.96 years, and 61.7 % were female.

**Table 2:** Food grouping used in the dietary pattern approach.

Food group	Food items
Whole grains	Barley, Sangak, Barbari, Corn and Groats
Refined cereals	Lavash, Taftoon, Baguette, Toast, Rice, Spaghetti, Vermicelli, Noodle and Wheat flour
Fruits	Cantaloupe, Melon, Canary melon, Watermelon, Pear, Apricot, Cherries, Apple, Peach, Nectarine, Greengage, Fresh fig, Grape, Kiwi, Grapefruit, Orange, Persimmon, Tangerine, Pomegranate, Plum, Strawberry, Banana, Sweat lemon, Lemon, Pineapple and fresh Berries
Vegetables	Lettuce, Tomato, Cucumber, Sabzi (mixed vegetable), Stewed vegetables, Green sqaush, Pumpkin, Eggplant, Celery, Green pea, Green bean, Carrot, Boiled carrots, Garlic, Onion, fried onions, Cabbage, Sweat pepper, Spinach, Stewed spinach, Turnip and Mushroom
Legumes	Lentil, bean, ChickPea, Split pea, Broad bean, Mung bean and Soybean
Nuts	Almond, walnut, pistachio, hazelnut, peanut, Sunflower seed and olive
Dried fruits	Raisin, Dried berry, Peach leaf, Apricot leaf, Date, Dried fig
Low-fat dairy products	Low-fat milk, cheese and low-fat yogurt, Dough(yogurt drink)
High-fat dairy products	High fat milk, Chocolate milk, Cream cheese, Strained yogurt, High fat yogurt, Creamy yogurt, Dough (yogurt drink), Curd (kashk), Traditional ice cream, Ice cream, Pizza cheese and Sour cream
White meat	Chicken with skin, Chicken without skin, All type of Fish, Tuna
Red meats and Organ meats	Beef, Lamb, Ground beef, Heart, liver, Kidney, tongue, brain, Tripe, Intestine, Viscera and Kale pache
Solid oil	Hydrogenated oils, rump, Animal fat, Butter and Margarine
Liquid oil	Liquid oil and olive oil
Eggs	Eggs
Pizza	Pizza
Snack	French fries, Chips and Puff
Processed meats	All type of Sausages, Kielbasa and Hamburger
Sweet drinks	Soft drinks, Apple juice, Orange juice, Cantaloupe juice and Compote
Sweets and Desserts	Biscuit, Cracker, Yazdi cake, Cake, Pastry, Cookie, Gaz, Candy, Sugar, Honey, Jam, Sohan, Chocolate, Caramel, Noghl, Rock candy, Halva and Doughnuts
Potato	Potato
Mayonnaise sauce	Mayonnaise sauce
Salt	Salt
Tea and coffee	Tea and Coffee

In the factor analysis, we identified four dietary patterns including “Western”, “unhealthy”, “traditional”, and “semi-healthy” (Supplementary Table 2). The “Western” dietary pattern was characterized by high consumption of snacks, potatoes, sweets and desserts, pizza, sweetened drinks, and egg. The “unhealthy” dietary pattern had a high content of red and organ meats, refined grains, solid fats, and a low amount of fruits, vegetables, legumes, and low-fat dairy products. The “traditional” dietary pattern was associated with high intakes of nuts, liquid oils, high-fat dairy products, mayonnaise sauce, and dried fruits. The last pattern was a “semi-healthy” dietary pattern characterized by high consumption of white meat, whole grains, processed meat, and a low salt intake. The four dietary patterns explained 34.29 % of the whole variance in dietary intakes.

Demographic characteristics and dietary intakes of participants across the tertiles of dietary pattern scores are shown in Table 3. We found a significant difference in age, gender, and marital status across the tertiles of the Western and unhealthy dietary patterns. BMI and diabetes-related training were different among the tertiles of traditional dietary pattern. In terms of dietary intakes, compared with those in the lowest tertile, individuals in the highest tertile of the Western diet had higher intakes of red meat, low-fat dairy, energy, protein, carbohydrate, fat, sodium, calcium, fiber, and a lower intake of nuts. Such the differences were observed for other dietary patterns.

**Table 3:** Demographic characteristics and Dietary intakes of Iranian T1D patients, aged ≥18 years, across tertiles of dietary patterns, 2017–2019.

	Tertiles of “Western” dietary pattern				Tertiles of “Unhealthy” dietary pattern				Tertiles of “Traditional” dietary pattern				Tertiles of “Semi-healthy” dietary pattern			
	T1	T2	T3	P*	T1	T2	T3	P*	T1	T2	T3	P*	T1	T2	T3	P*
*Demographic variables*																
Age (y)	35.0 ± 12.4	30.9 ± 9.6	30.9 ± 10.2	0.03	34.2 ± 11.9	32.8 ± 10.4	29.8 ± 10.1	0.04	33.3 ± 11.4	32.5 ± 10.1	30.9 ± 11.2	0.39	32.2 ± 12.4	32.1 ± 10.7	32.5 ± 9.7	0.97
BMI (kg/m^2^)	24.9 ± 3.7	25.0 ± 3.8	24.3 ± 3.5	0.51	25.0 ± 3.9	24.9 ± 3.5	24.4 ± 3.6	0.66	24.6 ± 3.4	26.6 ± 4.2	24.1 ± 3.3	0.05	24.6 ± 3.6	24.8 ± 4.3	24.8 ± 3.1	0.92
PA (MET-h/wk)	454 ± 357	486 ± 495	508 ± 480	0.76	535 ± 508	472 ± 379	441 ± 446	0.42	507 ± 458	488 ± 443	453 ± 444	0.75	392 ± 324	528 ± 467	527 ± 519	0.10
HbA1c (%)	8.0 ± 1.5	7.9 ± 1.5	8.3 ± 1.6	0.28	8.1 ± 1.9	8.2 ± 1.4	8.0 ± 1.3	0.67	7.8 ± 1.4	8.2 ± 1.6	8.3 ± 1.6	0.22	8.3 ± 1.6	8.0 ± 1.7	8.0 ± 1.4	0.53
Insulin dose (unit)	50.7 ± 15.5	51.4 ± 18.6	55.5 ± 18.8	0.20	49.5 ± 14.4	50.0 ± 17.8	58.1 ± 19.6	0.003	53.4 ± 22.1	52.9 ± 15.9	51.3 ± 14.6	0.76	53.7 ± 19.6	51.3 ± 15.1	52.6 ± 18.5	0.71
Female %	71.1	67.5	47.4	<0.01	63.2	74.0	48.7	<0.01	59.2	71.4	55.3	0.10	69.7	71.4	44.7	<0.01
Current smoker %	18.4	16.9	25.0	0.41	17.1	15.6	27.6	0.12	18.4	19.5	22.4	0.82	89.5	79.2	71.1	0.02
DM-related training (yes %)	85.5	80.5	85.5	0.62	88.2	84.4	78.9	0.30	93.4	81.8	76.3	0.01	76.3	89.6	85.5	0.07
Married %	56.6	35.1	38.2	0.01	42.1	49.4	38.2	0.36	40.8	48.1	40.8	0.57	43.4	44.2	42.1	0.96
University educated %	67.1	64.9	60.5	0.69	60.5	67.5	64.5	0.66	69.7	63.6	59.2	0.39	46.1	71.4	75.0	<0.01
DM duration (>10 y %)	72.4	63.6	71.1	0.45	78.9	68.8	59.2	0.03	71.1	76.6	59.2	0.06	69.7	71.4	65.8	0.74
Drug use (yes %) ^b^	28.9	26.0	15.8	0.13	23.7	27.3	19.7	0.54	26.3	26.0	18.4	0.43	27.6	27.3	15.8	0.14
Insulin type %				0.30				0.31				0.61				0.26
Lantus and NovoRapid	65.8	67.5	61.8		64.5	67.5	63.2		67.1	66.2	61.8		61.8	67.5	65.8	
NPH and Regular	25.0	13.0	26.3		17.1	22.1	25.0		18.4	20.8	25.0		27.6	19.5	17.1	
Lantus and Apidra	3.9	9.1	2.6		3.9	6.5	5.3		5.3	3.9	6.6		3.9	7.8	3.9	
Levemir and NovoRapid	5.3	3.9	5.3		7.9	0	6.6		5.3	6.5	2.6		2.6	2.6	9.2	

*Food intakes*																
Fruits	432.4 ± 354	392.8 ± 417	413.9 ± 255	0.78	592.0 ± 509	338.8 ± 170	308.9 ± 175	<0.01	286.2 ± 176	360.7 ± 268	592.6 ± 460	<0.01	330.5 ± 222	348.0 ± 207	561.1 ± 492	<0.01
Vegetables	562.4 ± 524	409.5 ± 284	521.6 ± 426	0.07	783.7 ± 580	408.6 ± 229	301.2 ± 177	<0.01	383.8 ± 347	426.6 ± 289	682.8 ± 541	<0.01	506.1 ± 452	441.7 ± 401	545.2 ± 422	0.31
Red meat	49.1 ± 35.0	63.5 ± 47.6	66.9 ± 53.4	0.04	44.8 ± 38.5	61.9 ± 42.8	72.9 ± 52.9	<0.01	43.3 ± 30.5	60.5 ± 42.2	75.8 ± 57.3	<0.01	47.7 ± 37.2	61.6 ± 51.8	70.4 ± 46.7	<0.01
Low-fat dairy	233.9 ± 170	318.9 ± 323	333.4 ± 253	0.03	449.7 ± 354	276.1 ± 146	160.9 ± 122	<0.01	342.1 ± 329	293.5 ± 209	251.0 ± 219	0.09	306.4 ± 322	285.0 ± 234	295.3 ± 213	0.87
Nuts	29.2 ± 62.7	13.0 ± 14.3	21.7 ± 14.9	0.03	31.0 ± 60.0	20.0 ± 24.9	12.9 ± 10.0	0.01	10.9 ± 6.9	21.3 ± 21.9	31.5 ± 61.2	<0.01	13.7 ± 11.1	15.1 ± 13.0	35.1 ± 62.6	<0.01
Whole grains	114.9 ± 96	109.2 ± 70	105.5 ± 78	0.77	107.1 ± 73	114.7 ± 93	107.7 ± 78	0.81	80.3 ± 62.4	113.7 ± 74.3	135.6 ± 97.0	<0.01	70.1 ± 53.2	100.3 ± 61.1	159.2 ± 98.5	<0.01

*Nutrient intakes*																
Energy (kcal)	2275 ± 754	2547 ± 682	3156 ± 614	<0.01	2764 ± 785	2471 ± 733	2744 ± 783	0.03	2139 ± 567	2584 ± 683	3254 ± 628	<0.01	2361 ± 784	2555 ± 724	3061 ± 646	<0.01
Protein (g/d)	82.0 ± 29.7	95.5 ± 25.4	121.5 ± 26	<0.01	109.6 ± 32.4	93.0 ± 29.3	96.4 ± 31.1	<0.01	87.5 ± 28.5	97.1 ± 30.6	114.4 ± 30.1	<0.01	81.9 ± 27.8	94.2 ± 25.5	122.8 ± 26.9	<0.01
CHO (g/d)	327.2 ± 121	359.8 ± 121	432.0 ± 98	<0.01	393.0 ± 129	347.3 ± 114	378.8 ± 119	0.06	295.7 ± 81	355.1 ± 107	468.2 ± 106	<0.01	331.4 ± 116	360.1 ± 109	427.4 ± 121	<0.01
Fat (g/d)	77.0 ± 32.1	86.2 ± 27.3	112.7 ± 31.7	<0.01	92.9 ± 34.3	84.1 ± 29.8	98.9 ± 36.0	0.02	71.6 ± 26.8	92.1 ± 30.5	112.1 ± 31.5	<0.01	84.4 ± 33.2	87.9 ± 35.6	103.5 ± 29.9	<0.01
Sodium (g/d)	5.6 ± 3.6	5.7 ± 3.1	7.7 ± 6.0	<0.01	7.9 ± 6.5	5.6 ± 2.7	5.4 ± 2.8	<0.01	5.6 ± 4.0	6.0 ± 3.3	7.4 ± 5.8	0.03	8.4 ± 6.5	5.0 ± 1.9	5.6 ± 3.1	<0.01
Calcium (g/d)	1.0 ± 0.4	1.2 ± 0.5	1.5 ± 0.4	<0.01	1.5 ± 0.6	1.1 ± 0.3	1.0 ± 0.3	<0.01	1.1 ± 0.5	1.2 ± 0.4	1.4 ± 0.4	<0.01	1.2 ± 0.6	1.2 ± 0.4	1.3 ± 0.4	0.05
Fiber (g/d)	24.6 ± 17.0	21.1 ± 10.9	27.0 ± 12.0	0.03	34.0 ± 18.4	20.4 ± 6.7	18.3 ± 6.4	<0.01	17.9 ± 6.9	21.3 ± 8.8	33.4 ± 17.6	<0.01	21.2 ± 10.8	21.4 ± 9.6	30.1 ± 17.5	<0.01

Data are presented as mean ± SD or percent.

Abbreviation: T: tertiles, BMI: body mass index, T1D: type 1 diabetes, DM: diabetes mellitus, PA: physical activity.

*Obtained from the analysis of variance (ANOVA) or chi-squared test, where appropriate.

^b^ Considered as the use of antihypertensive medication, lipid lowering medication and Levothyroxine.

Multivariable-adjusted ORs (95 % CIs) for the associations of dietary patterns with laboratory parameters, anthropometric measures, and blood pressure are shown in Table 4. After controlling for potential confounders, patients in the highest tertile of Western diet had 2.08 (OR: 2.08, 95 % CI: 1.01–4.28) and 2.50 (OR: 2.53, 95 % CI: 1.03–6.22) times more odds for having elevated FBG and HbA1c, respectively, compared with those in the lowest tertile. Such the positive association was also found for the low eGDR in the fully adjusted model; such that greater adherence to the Western diet was associated with 3.37 times higher odds for having low eGDR (OR: 3.37, 95 % CI: 1.18–9.63). Moreover, there was a significant positive association between the unhealthy diet and elevated LDL-c (OR: 2.17, 95 % CI: 1.02–4.60). A significant inverse association was seen between adherence to the semi-healthy diet and elevated FBG and TC after controlling for covariates (FBG: OR: 0.49, 95 % CI: 0.24–0.99, TC: 0.38, 95 % CI: 0.16–0.90). In terms of abdominal obesity, after controlling for potential confounders, adherence to the unhealthy dietary pattern was positively associated with abdominal obesity [obtained from WC (3.26, 95 % CI: 1.02–10.47) and WHR (4.22, 95 % CI: 1.42–12.57)]. Regarding general obesity (based on BMI and BFP) and elevated blood pressure, no significant association was found with dietary patterns in both crude and adjusted models.

**Table 4:** Multivariable ORs (95 % CIs) for the associations of dietary patterns with laboratory parameters, anthropometric measures, and blood pressure in Iranian T1D patients ages ≥18 years, 2017–2019.

	Tertiles of “Western” dietary pattern		Tertiles of “Unhealthy” dietary pattern		Tertiles of “Traditional” dietary pattern		Tertiles of “Semi-healthy” dietary pattern	
	T2	T3	T2	T3	T2	T3	T2	T3
*FBG > 130 mg/dL*								
Crude	0.78 (0.41–1.48)	2.26 (1.11–4.59)	0.69 (0.36–1.33)	1.19 (0.60–2.36)	1.28 (0.66–2.49)	1.05 (0.54–2.04)	1.02 (0.51–2.00)	0.67 (0.34–1.30)
Adjusted	0.78 (0.40–1.50)	2.08 (1.01–4.28)	0.71 (0.36–1.38)	1.06 (0.53–2.12)	1.26 (0.64–2.49)	0.96 (0.49–1.89)	0.91 (0.45–1.82)	0.49 (0.24–0.99)

*HbA1c > 7 %*								
Crude	1.49 (0.73–3.02)	2.17 (1.02–4.60)	1.33 (0.64–2.75)	1.31 (0.63–2.71)	0.95 (0.46–1.94)	1.23 (0.59–2.60)	0.48 (0.23–1.00)	0.85 (0.39–1.86)
Adjusted	1.46 (0.68–3.13)	2.53 (1.03–6.22)	1.36 (0.63–2.98)	1.32 (0.60–2.89)	0.54 (0.24–1.21)	0.56 (0.21–1.47)	0.55 (0.25–1.21)	1.10 (0.44–2.75)

*TG > 150 mg/dL*								
Crude	0.57 (0.13–2.49)	2.15 (0.69–6.62)	0.98 (0.30–3.20)	1.00 (0.30–3.25)	0.59 (0.18–1.89)	0.60 (0.19–1.92)	0.40 (0.10–1.60)	1.16 (0.39–3.37)

*TC > 200 mg/dL*								
Crude	1.11 (0.55–2.24)	0.64 (0.30–1.37)	1.20 (0.58–2.50)	1.15 (0.55–2.40)	1.33 (0.67–2.66)	0.49 (0.22–1.08)	1.33 (0.67–2.66)	0.49 (0.22–1.08)
Adjusted	1.15 (0.56–2.35)	0.62 (0.28–1.37)	1.19 (0.56–2.50)	1.08 (0.51–2.31)	1.28 (0.63–2.60)	0.46 (0.20–1.04)	1.17 (0.57–2.37)	0.38 (0.16–0.90)

*LDL-c > 100 mg/dL*								
Crude	0.56 (0.27–1.15)	1.00 (0.46–2.14)	1.30 (0.65–2.61)	2.17 (1.02–4.60)	1.16 (0.56–2.39)	1.06 (0.52–2.18)	0.94 (0.45–1.98)	0.71 (0.34–1.47)

*Low HDL-c ^a^*								
Crude	0.98 (0.48–1.97)	0.81 (0.39–1.67)	1.27 (0.62–2.60)	1.14 (0.55–2.36)	1.25 (0.63–2.48)	0.60 (0.28–1.28)	0.71 (0.34–1.44)	0.77 (0.38–1.56)
Adjusted	1.03 (0.49–2.17)	1.28 (0.58–2.81)	1.12 (0.52–2.39)	1.55 (0.71–2.40)	0.97 (0.46–2.02)	0.64 (0.29–1.42)	0.64 (0.30–1.36)	1.14 (0.53–2.48)

*Low eGDR ^b^*								
Crude	1.44 (0.71–2.91)	2.18 (1.09–4.35)	0.63 (0.31–1.27)	1.12 (0.57–2.16)	0.98 (0.50–1.91)	0.88 (0.45–1.74)	1.33 (0.67–2.66)	1.61 (0.81–3.20)
Adjusted	2.33 (0.91–5.98)	3.37 (1.18–9.63)	0.88 (0.35–2.24)	1.76 (0.72–4.31)	0.81 (0.33–1.97)	0.53 (0.19–1.47)	1.51 (0.63–3.64)	0.99 (0.38–2.61)

*WC > 90 cm*								
Crude	1.20 (0.58–2.50)	0.85 (0.40–1.84)	0.91 (0.42–1.94)	1.15 (0.55–2.40)	0.98 (0.47–2.02)	0.68 (0.32–1.47)	0.91 (0.42–1.94)	1.15 (0.55–2.40)
Adjusted	2.24 (0.75–6.68)	1.00 (0.29–3.46)	1.43 (0.43–4.79)	3.26 (1.02–10.47)	0.81 (0.27–2.38)	0.75 (0.25–2.28)	0.65 (0.21–2.04)	0.78 (0.25–2.48)

*High WHR ^c^*								
Crude	1.27 (0.56–2.86)	0.82 (0.34–1.96)	1.65 (0.66–4.09)	2.14 (0.88–5.17)	1.62 (0.72–3.66)	0.80 (0.32–2.00)	1.07 (0.46–2.47)	1.00 (0.43–2.32)
Adjusted	1.77 (0.68–4.59)	1.10 (0.41–2.96)	2.19 (0.76–6.33)	4.22 (1.42–12.57)	1.49 (0.60–3.73)	0.84 (0.31–2.31)	1.25 (0.48–3.29)	1.11 (0.43–2.87)

*Elevated BP ^d^*								
Crude	0.32 (0.13–0.79)	0.74 (0.35–1.58)	0.66 (0.27–1.59)	1.58 (0.73–3.42)	0.82 (0.36–1.87)	1.08 (0.49–2.38)	0.90 (0.40–2.02)	1.00 (0.45–2.22)
Adjusted	0.25 (0.10–0.67)	0.48 (0.20–1.12)	0.81 (0.32–2.06)	1.49 (0.65–3.44)	0.82 (0.34–2.01)	1.09 (0.47–2.52)	0.86 (0.36–2.06)	0.60 (0.25–1.47)

*High BFP ^g^*								
Crude	1.03 (0.54–1.95)	0.59 (0.30–1.16)	1.36 (0.71–2.61)	1.05 (0.54–2.05)	2.09 (1.08–4.02)	0.94 (0.47–1.85)	1.09 (0.56–2.11)	1.32 (0.68–2.53)
Adjusted	1.02 (0.54–1.95)	0.59 (0.30–1.15)	1.38 (0.72–2.64)	1.08 (0.55–2.09)	2.10 (1.09–4.05)	0.95 (0.48–1.88)	1.08 (0.55–2.09)	1.30 (0.67–2.51)

*BMI > 25*								
Crude	1.41 (0.74–2.67)	1.11 (0.58–2.11)	0.83 (0.44–1.57)	0.85 (0.45–1.61)	1.26 (0.67–2.38)	0.64 (0.33–1.23)	0.79 (0.41–1.49)	1.05 (0.55–1.99)
Adjusted	1.64 (0.85–3.17)	1.28 (0.66–2.48)	0.87 (0.45–1.65)	0.97 (0.50–1.87)	1.30 (0.68–2.47)	0.69 (0.36–1.33)	0.78 (0.41–1.50)	1.04 (0.54–1.99)

Data are presented as OR (95 % CI) and patients in T1 category were considered as reference group.

Abbreviation: T: tertile, FBG: fasting blood glucose, TG: triglyceride, TC: total cholesterol, LDL-c: low-density lipoprotein cholesterol, HDL-c: high-density lipoprotein cholesterol, eGDR: estimated glucose disposal rate, T1D: type 1 diabetes, BMI: body mass index, WC: waist circumference, WHR: waist-to-hip ratio, BP: blood pressure, BFP: body fat percentage, BMI: body mass index, HbA1c: hemoglobin A1c.

^a^ Considered as serum HDL-c levels of <40 mg/dL in males and <50 mg/dL in females.

^b^ The first tertile of eGDR was considered as low eGDR.

^c^ Considered as WHR of >0.95 in males and >0.90 in females.

^d^ Considered as systolic blood pressure ≥135 and diastolic blood pressure ≥85.

^g^ Considered as BFP of >25 % in males and >39 % in females.

Adjusted model: the variables adjusted in this model are shown in Supplementary Table 1. For TG and LDL-c, we found no potential confounders based on the P threshold of 0.10 in the multiple logistic regression.

## 4 Discussion

In the current study, we found that adherence to the Western dietary pattern was associated with increased odds of elevated FBG, HbA1c and low eGDR. These results were in line with a cross-sectional study in which adherence to a “baked dessert” diet, similar to the Western diet in our study, was associated with elevated HbA1c in American T1D patients (Basu et al., 2021). Furthermore, some studies reported that adherence to a “soft drinks” diet that was rich in refined grains, sweets, chocolates, and sweetened beverages was associated with abnormal glucose homeostasis and insulin resistance (Panagiotakos et al., 2005, Kahn and Tatham, 1997). In the current study, the Western diet contained dietary sources of saturated fatty acid (SFA) such as processed meat, mayonnaise, and high-fat dairy products. It is well-known that SFA in the bloodstream impairs insulin signaling in non-adipose tissues, which causes whole-body insulin resistance (Kennedy et al., 2009). Moreover, the Western diet was rich in refined sugar sources including sweetened drinks, cookies, and desserts. It has long been shown that a high intake of refined sugar is associated with insulin resistance and abnormal glucose homeostasis (Valle et al., 2020). In total, T1D patients may benefit from a diet with a low amount of SFA and refined sugar sources.

Furthermore, we found a positive association between the unhealthy dietary pattern. and elevated LDL-c in T1D patients. In agreement with our findings, in a cross-sectional study in China, Jaacks et al. reported that adherence to a dietary pattern rich in wheat products and high-fat cakes was associated with increased levels of LDL-c (Jaacks et al., 2015). In another cross-sectional study on Iranian healthy individuals, having a Western-style diet (rich in sugar, pizza, and poor in fruits and vegetables) was associated with elevated levels of LDL-c (Asadi et al., 2020). Furthermore, in a cross-sectional study that was performed on 169 females, after adjustment for age, BMI, and energy intake, subjects who were in the highest tertile of FV pattern that was characterized with high intake of the fruits and vegetables had significantly lower levels of LDL‑c, triglyceride (TG), and total cholesterol (Sajjadpour et al., 2021). In contrast, a cohort study in Sweden showed no significant association between a Western diet (with high intakes of sugar-sweetened beverages and red and processed meats) and LDL-c levels in adults (Drake et al., 2018). The observed controversy might be explained by different health conditions of participants among previous studies. Also, considering different food items for unhealthy dietary patterns and different adjustments for confounders are other reasons for the controversy. Further studies are needed to confirm our findings on T1D patients.

The unhealthy dietary pattern in the present study contained a high amount of cholesterol- and SFA-rich foods such as processed meat, red meat, and mayonnaise. Based on the previous studies, dietary SFA can increase the production of endogenous cholesterol. Also, dietary cholesterol is positively correlated with LDL-c levels. In addition, SFA can inhibit LDL-c receptor activity and increase the production of apolipoprotein B-containing lipoprotein (Siri-Tarino et al., 2010).

We also observed an inverse association between the semi-healthy dietary pattern and elevated FBG and TC in T1D patients. We are aware of no study that examined the link between semi-healthy dietary patterns and blood glucose in T1D patients. However, previous studies have assessed this association in the general population and individuals with different health conditions. In a cross-sectional study on 10,741 US adults, adherence to a Dietary Approaches to Stop Hypertension (DASH) diet was inversely associated with blood glucose (Joyce et al., 2019). In another similar study on 1837 Malaysian and 2898 Philippines participants, Tiong et al. did not find any significant association between a healthy diet (containing fruits, vegetables, legumes, nuts, and whole grains) and blood glucose (Tiong et al., 2018). Different findings from the previous studies might be due to different study designs, different cooking processes of foods in different cultures, and controlling for different confounders among previous studies. In terms of anthropometric measures, we found a significant positive association between the unhealthy dietary pattern and abdominal obesity, but not general obesity, in T1D patients. The unhealthy diet contained a high amount of pizza, refined grains, sweet drinks, processed meat, red meat, mayonnaise sauce, solid oils, and a low amount of vegetables, legumes, low-fat dairy, and fruits. A cross-sectional study on Iranian adults showed a positive association between red meat consumption and abdominal obesity. However, this association was not significant for general obesity (Dabbagh-Moghadam et al., 2017). Adherence to a unhealthy diet is associated with a high intake of SFA and calorie-dense foods. Calorie-dense foods provide a high amount of energy that is stored as fat in abdominal fat tissue. Also, it has been shown that SFA intake has a more obesogenic effect compared with unsaturated fatty acids (Hariri et al., 2010). The lack of significant association between the unhealthy diet and general obesity might be explained by the fatty acid composition of this diet (rich in SFA and poor in unsaturated fatty acids) (Alsharari et al., 2017).

For blood pressure, we found no significant association with identified dietary patterns. In contrast to our result, the FinnDiane study showed that adherence to a diet rich in fresh vegetables, fruits, berries, cooked vegetables, fish dishes, and yogurt was associated with lower SBP and DBP in T1D (Ahola et al., 2016). Another study showed that having a Mediterranean diet was associated with lower blood pressure in T1D (Gingras et al., 2015). The lack of significant association in the current study might be attributed to a low number of patients with elevated blood pressure (9.6 %). Also, patients in the current study had lower age and this might be another reason for the lack of significant association.

The strengths of our study were the assessment of several cardiometabolic risk factors in T1D patients and their associations with major dietary patterns. Moreover, we used a validated instrument for dietary assessment. Furthermore, we tried to obtain an independent association between dietary patterns and cardiometabolic risk factors by controlling for several confounders. Despite the strengths, some limitations should be considered when interpreting our findings. Because of the cross-sectional nature of this study, we cannot establish a causal link between dietary patterns and cardiometabolic risk factors. Although a validated FFQ was used to assess dietary intakes in our study, the closed-end response nature of the questionnaire might increase the rate of misclassification. In addition to the misclassification, diabetic patients may present unreal information about past drug use and physical activity (social desirability bias). However, we tried to assess the drugs used by diabetic patients in the interview sessions. In addition, we used a validated questionnaire for physical activity assessment. Nevertheless, we cannot reject the possibility of over- or under-reporting physical activity. Another limitation was the low sample size of our study. However, this sample size had sufficient power to detect possible associations. Although several confounders were adjusted to assess the associations, further controlling for other variables such as supplement use and psychological disorders might be needed to reach an independent association.

In conclusion, we found a positive association between the Western dietary pattern and elevated FBG, HbA1c and low eGDR. Also, adherence to the unhealthy diet was positively associated with elevated LDL-c and abdominal obesity. In addition, a significant inverse relationship was observed between the semi-healthy dietary pattern and FBG and TC. In total, to prevent cardiometabolic risk factors in T1D patients, adherence to a diet with a high amount of whole grains, white meat, fruits, vegetables, legumes, low-fat dairy products, and a low amount of snacks, potatoes, sweets and desserts, pizza, sweetened drinks, salty foods, red and organ meats, refined grains, and solid fats is recommended. However, to confirm this claim, future clinical trials should examine the effect of this diet on cardiometabolic risk factors in T1D patients.

## CRediT authorship contribution statement

**Zahra Shojaeian:** Conceptualization, Data curation, Formal analysis, Funding acquisition, Investigation, Methodology, Resources, Software, Validation, Visualization, Writing original draft. **Zohreh Ebrahimi:** Conceptualization, Data curation, Formal analysis, Investigation, Methodology, Software. **Fatemehsadat Amiri:** Conceptualization, Data curation, Investigation, Methodology, Project administration, Supervision, Validation, Visualization, Writing-review & editing. **Ahmad Esmaillzadeh:** Formal analysis, Methodology, Validation. **Omid Sadeghi:** Formal analysis. **Seyed Adel Jahed:** Data curation, Investigation. **Alireza Esteghamati:** Data curation. **Ali Ebrahimkhani:** Data curation.

## Declaration of competing interest

The authors declare that they have no known competing financial interests or personal relationships that could have appeared to influence the work reported in this paper.

## Data Availability

Data will be made available on request.
