# Supplementary material for: Associations of major dietary patterns with cardiometabolic risk factors among Iranian patients with type 1 diabetes
Source: Prev Med Rep. 2024 Jan 18;38:102618. doi: 10.1016/j.pmedr.2024.102618 (PMC10874838; doi:10.1016/j.pmedr.2024.102618)
Supplement: Supplementary data 1 [file mmc1.docx]

**Supplementary Table 1**: The variables adjusted in the associations of dietary patterns with laboratory parameters, anthropometric measures, and blood pressure in Iranian T1D patients ages ≥18 years, 2017-2019

| **Dependent variables** | **Confounders** |
| --- | --- |
| FBG>130 mg/dL | Smoking, drug use |
| HbA1c>7% | DM-related training, gender, physical activity, energy intake, drug use |
| TG>150 mg/dL | None |
| TC>200 mg/dL | Smoking, gender |
| LDL-c>100 mg/dL | None |
| Low HDL-c ^a^ | Gender, BMI |
| Low eGDR ^b^ | Age, gender, energy intake, duration of diabetes, drug use, BMI |
| WC>90 cm | Age, gender, BMI, duration of diabetes |
| High WHR ^c^ | Age, physical activity, BMI |
| Elevated BP ^d^ | Sex, BMI |
| High BFP ^g^ | physical activity |
| BMI>25 | Age |

Abbreviation: FBG: fasting blood glucose, TG: triglyceride, TC: total cholesterol, LDL-c: low-density lipoprotein cholesterol, HDL-c: high-density lipoprotein cholesterol, eGDR: estimated glucose disposal rate, T1D: type 1 diabetes, BMI: body mass index, WC: waist circumference, WHR: waist-to-hip ratio, BP: blood pressure, BFP: body fat percentage, BMI: body mass index, HbA1c: hemoglobin A1c

^a^ Considered as serum HDL-c levels of <40 mg/dL in males and <50 mg/dL in females

^b^ The first tertile of eGDR was considered as low eGDR

^c^ Considered as WHR of >0.95 in males and >0.90 in females

^d^ Considered as systolic blood pressure ≥135 and diastolic blood pressure ≥85

^g^ Considered as BFP of >25% in males and >39% in females

**Supplementary Table 2**: Food loading matrix for major dietary patterns

| Food group | Dietary patterns | | | |
| --- | --- | --- | --- | --- |
|  | “Western” | “Unhealthy” | “Traditional” | “Semi-healthy” |
| Potato | 0.532 |  |  |  |
| Sweets and desserts | 0.515 |  |  |  |
| Pizza | 0.472 |  |  |  |
| Sweetened drinks | 0.428 |  |  |  |
| Eggs | 0.422 |  |  |  |
| Vegetables |  | - 0.596 |  |  |
| Legumes |  | - 0.559 |  |  |
| Low-fat dairy products |  | - 0.506 |  |  |
| Fruits |  | -0.468 |  |  |
| Refined grains |  | 0.409 |  |  |
| Red meats & Organ meats |  | 0.287 |  |  |
| Solid fats |  | 0.212 |  |  |
| **Dried fruits** |  | ­­ | 0.608 |  |
| Liquid oils |  |  | 0.551 |  |
| High-fat dairy products |  |  | 0.457 |  |
| Mayonnaise sauce |  |  | 0.410 |  |
| Nuts |  |  | 0.349 |  |
| White meats |  |  |  | 0.803 |
| Whole grains |  |  |  | 0.451 |
| Processed meats |  |  |  | 0.429 |
| Salt |  |  |  | - 0.418 |
| Tea and coffee |  | - | - | - |
| Variance explained (%) | 10.02 | 9.16 | 7.58 | 7.52 |

The highest factor loadings values were remained for simplicity.
